# Rationale and emerging evidence for microglial replacement in Alzheimer’s disease

**DOI:** 10.1016/j.mocell.2025.100265

**Published:** 2025-08-14

**Authors:** Jee Yoon Bang, Yongjin Yoo

**Affiliations:** 1 Department of Neuroscience, Korea University College of Medicine, Seoul, Republic of Korea; 2 Picower Institute for Learning and Memory, Massachusetts Institute of Technology, Cambridge, MA, USA; 3 Department of Brain and Cognitive Sciences, Massachusetts Institute of Technology, Cambridge, MA, USA

**Keywords:** Alzheimer's disease, Cell therapy, Microglia, Microglia replacement, Neurodegenerative disease

## Abstract

Microglial biology in Alzheimer’s disease (AD) has become a major focus of investigation, aiming to define how these cells contribute to neurodegeneration and to develop new therapeutic strategies. Once regarded as passive responders, microglia are now recognized as active regulators of brain homeostasis, immune signaling, and synaptic remodeling. Their interactions with genetic risk variants and age-related changes are increasingly understood to play central roles in AD pathogenesis. In this mini-review, we summarize recent progress in identifying microglial contributions to AD through genetic and transcriptomic studies. We discuss how microglia respond to amyloid-β and tau pathology by shifting into diverse functional disease-associated states, which may either protect or harm the brain depending on context and disease stage. We also outline the rationale for targeting microglia through replacement strategies and review emerging approaches using circulation-derived myeloid cells (CDMCs), and human pluripotent stem cell–derived microglia-like cells. These replacement methods have shown potential to rectify microglial functions and modify AD-related pathology in preclinical models, offering a novel therapeutic direction for neurodegenerative diseases.

## INTRODUCTION

Microglia are specialized, tissue-resident phagocytic cells of the central nervous system (CNS) that are indispensable for brain development, homeostasis, and immune response (Prinz et al., 2019, Salter and Stevens, 2017). Unlike neurons and macroglia, derived from neuroectodermal progenitors, microglia originate from yolk sac (YS)–derived erythromyeloid precursors that colonize the developing brain before the establishment of the blood-brain barrier (Masuda et al., 2020b). These precursors give rise to long-lived, self-renewing populations that persist independently of postnatal hematopoiesis (Davalos et al., 2005; Ginhoux et al., 2010; Goldmann et al., 2016; Kierdorf et al., 2013; Li and Barres, 2018; Mass et al., 2023; Nimmerjahn et al., 2005).

Contrary to previous notions of microglia as quiescent under physiological conditions, these cells are diverse and exhibit dynamic surveillance capabilities. Through their motile processes, microglia constantly assess the microenvironment, allowing for responses to injury, infection, or neurodegeneration (Mass et al., 2023; Nimmerjahn et al., 2005). Activation induces morphological and transcriptional changes, including process retraction, enhanced motility, localized proliferation, and the secretion of various cytokines, chemokines, and neuroactive molecules. These responses span a phenotypic continuum rather than fitting a simple “resting” versus “activated” dichotomy (Paolicelli et al., 2022, Ransohoff and El Khoury, 2015). Multiple studies have demonstrated the extensive functional plasticity of microglia, including their ability to phagocytose apoptotic cells and cellular debris, produce proinflammatory mediators (eg, TNF-α, IL-1β, and IL-6) that influence synaptic strength and immune tone (Biber et al., 2007, Liu et al., 2016), and subsequently release factors (eg, IL-10, TGF-β, and IGF-1) that facilitate tissue repair and neurogenesis (Butovsky et al., 2014, Nakanishi et al., 2021, Sierra et al., 2010).

Furthermore, microglia can integrate signals from neurons, astrocytes, and peripheral immune components via diverse membrane receptors and ion channels. For example, the purinergic receptor *P2RY12* mediates microglial chemotaxis toward ATP/ADP released by stressed or active neurons (Haynes et al., 2006, Mildner et al., 2016), while the CX3C motif chemokine receptor 1 (CX3CR1), interacting with neuronal fractalkine (CX3CL1), regulates synaptic pruning and promotes anti-inflammatory states (Paolicelli et al., 2011, Zhan et al., 2014). Triggering receptor expressed on myeloid cells 2 (TREM2), a lipid-sensing receptor critical in disease contexts, facilitates metabolic adaptation and the establishment of disease-associated microglial (DAM) phenotypes (Deczkowska et al., 2018, Keren-Shaul et al., 2017, Ulland et al., 2017). These receptor interactions enable microglia to decode molecular environments and modulate their responses.

Beyond immune surveillance, microglia play a role in CNS development and synaptic plasticity. Through complement-mediated pruning, microglia refine neuronal circuits by phagocytosing surplus synapses tagged by neuronal C1q and C3, recognized by microglial complement receptor 3 (CR3/CD11b) (Schafer et al., 2012, Stevens et al., 2007). Dysregulation of this process has been implicated in various neurological disorders, from neuropsychiatric disorders to neurodegenerative diseases (Hong et al., 2016, Sellgren et al., 2019). Additionally, microglia modulate neurotransmission, dendritic spine formation, and astrocyte functionality, thus playing integral roles within the tripartite synapse (Prinz et al., 2019). These diverse functions are rooted in the conserved developmental origin of microglia and their evolutionarily preserved roles in CNS homeostasis and injury response across species (Kierdorf and Prinz, 2013, Prinz et al., 2019).

Due to their roles in maintaining CNS integrity and involvement in disease states, microglia represent targets for replacement therapies (Gao et al., 2023, Guan et al., 2025, Rao and Peng, 2024, Walsh and Lukens, 2025). Dysfunctional microglial responses contribute to pathological conditions such as Alzheimer's disease (AD), multiple sclerosis, amyotrophic lateral sclerosis, Parkinson’s disease, Huntington’s disease, and traumatic brain injuries. Recent studies highlight microglial transplantation and rejuvenation strategies in preclinical models and underscore the potential of targeting microglia to restore homeostasis, alleviate neuroinflammation, and improve outcomes in neurological disorders. Therefore, understanding and harnessing microglial biology represents a frontier in developing therapeutic paradigms. In this mini-review, we explore microglial biology in the context of neurodegenerative disorders, with a focus on AD. We highlight the rationale for targeting microglia in cell-based therapeutic strategies, review key studies employing diverse approaches, and discuss future directions, including current knowledge gaps.

## MICROGLIA IN ALZHEIMER’S DISEASE

### Genetics/Epigenetics and AD Microglia

AD is a progressive neurodegenerative disease with debilitating symptoms, including memory loss, personality changes, and impaired executive functions. AD exists in both familial and sporadic forms, with the latter accounting for the vast majority of cases. Familial AD, driven by rare autosomal-dominant mutations in genes, such as *APP*, *PSEN1*, and *PSEN2*, has provided key mechanistic insights into amyloid and tau pathology (Scheltens et al., 2021, Selkoe and Hardy, 2016).

Most AD cases are late-onset and sporadic, arising from a complex interplay of genetic predispositions, environmental exposures, and aging. These factors are thought to disrupt multiple aspects of brain homeostasis, including the clearance of β-amyloid (Aβ) and regulation of tau phosphorylation, ultimately contributing to plaque and tangle formation, chronic neuroinflammation, and eventual neuronal loss (Frisoni et al., 2022, Korczyn and Grinberg, 2024, Long and Holtzman, 2019, Mawuenyega et al., 2010).

**Fig. 1:**
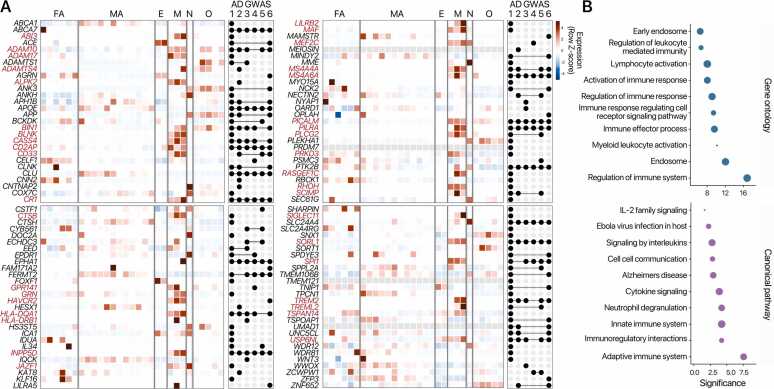
Alzheimer's disease (AD)-associated genes with cell-type-specific expression and its functions. (A) Among 112 AD risk genes, 38 (indicated in red) demonstrated significant expression in human brain microglia. (B) These AD risk genes are notably associated with microglia-mediated innate immune functions, including immune response and cytokine signaling. Curated AD risk genes were obtained from Andrews et al. (2023). Their expression in microglia was assessed by Student's t-test using normal human brain RNA-seq datasets (Zhang et al., 2016). Functional enrichment analysis for Gene Ontology terms and pathways was performed using mSigDB (Mootha et al., 2003;Subramanian et al., 2005;Liberzon et al., 2015). FA, fetal astrocytes; MA, mature astrocytes; E, endothelial cells; M, microglia; N, neurons; O, oligodendrocytes. AD GWAS studies indicate 1: Bellenguez et al. (2022), 2: Jansen et al. (2019), 3: Kunkle et al., (2020), 4: Lambert et al. (2013), 5: Marioni et al. (2018), 6: Wightman et al., 2022. GWAS, genome-wide association studies.

Accordingly, genome-wide association studies over the past decade have identified more than 70 genetic loci associated with late-onset AD, many involving genes selectively or predominantly expressed in microglia, such as *TREM2*, *APOE*, *CD33*, *INPP5D*, *ABI3*, and members of the *MS4A* gene clusters (Bellenguez et al., 2022, Kunkle et al., 2019, Lambert et al., 2013, Wightman et al., 2021). Many of these risk genes show cell-type-specific enrichment in human microglia (Fig. 1A) and are functionally associated with pathways involved in immune activation, cytokine signaling, and neuroinflammatory processes (Fig. 1B). To understand how these variants affect gene regulation, studies have mapped chromatin accessibility and enhancer-promoter interactions in brain cell types, revealing that AD risk variants are enriched in microglia-specific regulatory elements, particularly in regions bound by lineage-defining transcription factors such as *PU.1*, which orchestrate microglial gene expression programs (Corces et al., 2020, Kosoy et al., 2022, Nott et al., 2019, Novikova et al., 2021).

Furthermore, complementing insights from regulatory elements, rare coding mutations in *TREM2*, such as the R47H variant, highlight the importance of microglial genes in AD pathogenesis. These mutations impair microglial responses to amyloid and lipid metabolism and confer a 3- to 4.5-fold increased risk of AD, comparable to the risk associated with a single copy of *APOE ε4* (Guerreiro et al., 2013, Ulland et al., 2017). In addition, Gjoneska et al. (2015) showed that enhancers activated in immune-related genes during AD pathology are similarly upregulated in both human brain and AD mouse models. These cross-species observations strengthen the case for using preclinical systems to dissect the impact of genetic risk on microglial biology, while also suggesting that key regulatory signatures are stable and potentially therapeutically targetable.

### Transcriptional Heterogeneity of Microglia in AD

**Fig. 2:**
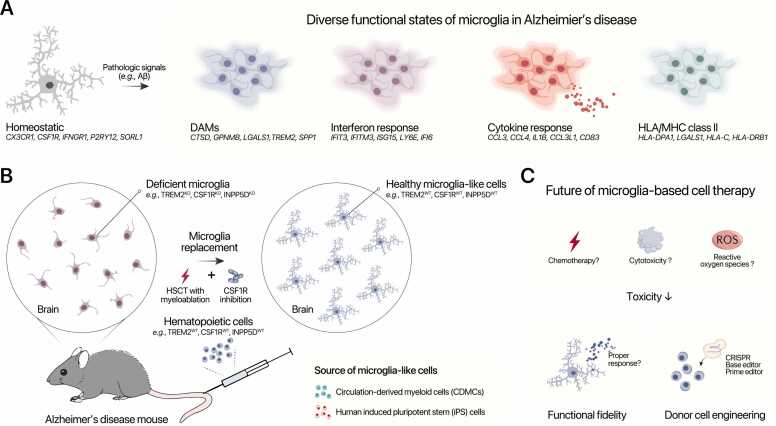
Microglial state diversity, replacement strategies, and therapeutic directions in Alzheimer's disease. (A) Under homeostatic conditions, microglia express markers such as *CX3CR1*, *P2RY12*, and *TREM2*. In response to pathogenic stimuli [eg, amyloid beta (Aβ)], they transition into diverse disease-associated states, including disease-associated microglia (DAMs), interferon-response microglia, cytokine-response microglia, and MHC class II–expressing microglia. Each state is characterized by a distinct transcriptional profile (Fumagalli et al., 2025). (B) Microglial replacement in Alzheimer's disease models involves depleting dysfunctional or genetically deficient microglia (eg, *TREM2*^*−/−*^*CSF1R*^*−/−*^) via myeloablation and CSF1R inhibition, followed by engraftment of donor-derived microglia-like cells, circulation-derived myeloid cells, or human iPSCs. Transplanted cells can repopulate the brain and adopt microglial-like morphology. (C) Future directions for microglial cell therapy focus on improving engraftment efficiency and minimizing toxicity (eg, via oxidative stress reduction), enhancing functional fidelity, and applying gene-editing strategies to generate disease-resistant donor cells. CSF1R, colony-stimulating factor 1 receptor; iPSC, induced pluripotent stem cell.

In the presence of Aβ and tau pathology, microglia transition into a DAM state, marked by increased expression of genes such as *Trem2*, *Apoe*, *Clec7a*, *Cst7*, and *Itgax*, and concurrent downregulation of homeostatic markers like *Tmem119* and *P2ry12* (Keren-Shaul et al., 2017, Krasemann et al., 2017, Wang et al., 2022). In addition to DAMs, recent single-cell and single-nucleus transcriptomic studies have revealed the emergence of diverse microglial states in AD, including interferon-response microglia (eg, expressing *Ifitm3*, *Isg15*), cytokine-response microglia (eg, secreting CCL2, IL-1β, and CCL3), and MHC class II–expressing microglia (eg, HLA-DRA, CD74), each reflecting unique transcriptional programs and immune reactivity (Fig. 2A) (Grubman et al., 2020, Masuda et al., 2020, Olah et al., 2020).

While this phenotypic shift initially confers protective effects by enhancing aggregate clearance and limiting toxicity, prolonged activation leads to dysfunctional states associated with impaired phagocytosis, lipid accumulation, metabolic dysregulation, and chronic inflammatory cytokine production (Marschallinger et al., 2020, Rachmian et al., 2024).

However, despite extensive transcriptomic profiling using single-nucleus RNA sequencing, findings across AD cohorts have revealed considerable heterogeneity and occasional inconsistencies in microglial signatures (Mathys et al., 2017, Mathys et al., 2019, Prater et al., 2023, Sun et al., 2023). These discrepancies likely reflect differences in disease stage, brain region, sample processing, and genetic background, and highlight the need for integrative approaches to resolve context-specific microglial states in AD.

### Compensatory Roles of Peripheral Myeloid Cells for Microglial Dysfunction in AD

In AD, conditions of chronic neuroinflammation and prolonged DAM state, exacerbated by the accumulation of senescent proteins in the brain environment, can recruit peripheral myeloid cells into the CNS from the circulation (Abellanas et al., 2025, Goldmann and Prinz, 2013, Muñoz-Castro et al., 2023, Goldmann et al., 2016). These myeloid cells originate from hematopoietic stem cells (HSCs) in the bone marrow and may retain functional plasticity and immunological “youth,” referring to reduced prior exposure to chronic neuroinflammation, preserved homeostatic responses, and a greater capacity to adopt to CNS cues compared with aged, disease-exposed microglia in AD. It is thought that upon infiltration, they adopt reparative phenotypes, characterized by the secretion of anti-inflammatory cytokines such as IL-10, TGF-β, and the expression of neurotrophic and immunomodulatory molecules, and contribute to the clearance of amyloid beta and cellular debris (Abellanas et al., 2025, Colella et al., 2024, Dvir-Szternfeld et al., 2022, Shemer et al., 2018, Varvel et al., 2012, Greenhalgh et al., 2018). Recent studies further reveal that skull bone marrow contributes a distinct pool of myeloid cells capable of migrating directly into the CNS, with region-specific inflammatory signatures observed in human AD brains (Cugurra et al., 2021, Du et al., 2024, Herisson et al., 2018). Upon CNS entry, these cells exhibit context-dependent phenotypes, adopting immunoregulatory traits in some settings, while displaying increased proinflammatory activity in others, particularly under chronic or autoimmune conditions. Their functional role in AD remains to be fully defined.

### Cell Therapy Approach to Target Microglia of AD

Substantial investigation linking microglial dysfunction to core features of AD pathology has provided a strong rationale for microglial replacement as a potential therapeutic strategy. In contrast to traditional approaches such as antibody-based or small-molecule treatments, which often face challenges like limited penetration into the CNS and short-lived effects, cell-based therapies offer the possibility of sustained benefit from a single intervention. Furthermore, enzyme replacement therapy has shown very limited efficacy due to severe constraints in tissue accessibility, particularly within the brain, and the short half-life of enzymes (Clarke et al., 2007, Sonoda et al., 2018, Schiffmann et al., 2001). In contrast, microglia replacement, which efficiently delivers healthy cells via the microglia niche generated by colony-stimulating factor 1 receptor (CSF1R) inhibition, may offer a valuable therapeutic strategy for various neurological diseases.

Originally developed for treating hematological malignancies, cell therapy is now being adapted for the brain, informed by progress in bone marrow transplantation and stem cell biology (Rash et al., 2025). Although microglial replacement has not yet been tested in humans for AD, related approaches such as HSCs transplantation (HSCT) have shown sustained clinical benefit in disorders like X-linked adrenoleukodystrophy and Hurler syndrome (Biffi et al., 2013, Cartier et al., 2009). These precedents support the feasibility of microglial replacement in the human brain and its therapeutic promise for AD. As described below, the studies of necessary conditions for microglial replacement and transplantable cell sources, and multiple preclinical studies have demonstrated phenotypic alleviations in AD mouse models through the replacement of defective microglia with transplanted donor cells (Table 1). For instance, transplantation of CDMCs, acting as microglia-like cells, contributed to the amelioration of Aβ pathology in the preclinical mouse model of AD by engaging in Aβ plaque phagocytosis, reducing amyloid plaque deposition, and promoting Aβ plaque compaction (Yoo et al., 2023) (Table 1).

**Table 1:** Therapeutic applications of microglia replacement in early- and late-onset neurodegenerative diseases

Disease	Donor cells	Injection	Preconditioning	CSF1R inhibition	Model	Benefits	References
AD	Whole–bone marrow cells	RO	Busulfan	PLX5622	Trem2−/− 5xFAD	• Recovery of cellular barrier around plaques • Reduction in Aβ deposition • Reduction in LAMP1+ dystrophic neurites	Yoo et al. (2023)
AD	Hematopoietic stem and progenitor cells	IV	Irradiation	-	5xFAD	• Reduction in neuroinflammation • Reduction in Aβ deposition • Improved memory	Mishra et al. (2023)
AD	Aβ CAR-Ms	ICV	Busulfan	PLX5622	APP/PS1 mice	• Functional assessment of CAR-Ms, including plaque phagocytosis and degradation • CAR-Ms with elevated CSF1 expression demonstrated increased phagocytosis of plaques	Kim et al.(2024)
Granulin FTD	Whole–bone marrow cells	IV, ICV	Busulfan	PLX3397	Grn−/−	• Correction of lipofuscin accumulation • Recovery from defects in proteostasis and lipid metabolism	Colella et al. (2024)
CSF1R-ALSP	Whole–bone marrow cells	IV	Busulfan	PLX5622	Csf1r-WT/I792T or Csf1r-WT/E631K	• Suppressed brain pathology • Normalized neural signal transduction • Ameliorated motor deficits	Wu et al. (2025)
SD	Whole–bone marrow cells	RO	Irradiation	PLX5622	Hexb−/−	• Reversed expression of apoptosis-associated genes • Resolution of glycolipid/glycoprotein storage • Clearance of accumulated lysosomal components • Reduction of vacuolization	Tsourmas et al. (2024)

Aβ, amyloid beta; AD, Alzheimer’s disease; ALSP, adult-onset leukoencephalopathy with axonal spheroids and pigmented glia; CAR-M, chimeric–antigen receptor macrophages; FTD, frontotemporal dementia; ICV, intracerebroventricular; IV, intravenous; RO, retro-orbital; SD, Sandhoff disease; WT, wild type.

### Microglia-free Niche via CSF1R Inhibition

Strategies to deplete microglia and facilitate donor cell engraftment have been instrumental in evaluating microglial replacement in AD models. In preclinical mouse models of AD, microglial replacement is achieved by conditioning the brain through CSF1R inhibition and/or irradiation, followed by transplantation of hematopoietic or CDMCs, which can engraft and occupy the vacated niche (Fig. 2B).

The most widely used approach involves pharmacological inhibition of CSF1R, a receptor essential to survival of microglia. Small-molecule inhibitors such as PLX5622 and PLX3397, typically delivered via chow, can eliminate 90% to 99% of microglia within 1 to 3 weeks in adult mice (Dagher et al., 2015, Elmore et al., 2014, Spangenberg et al., 2016). Less commonly, localized depletion has been achieved via intraperitoneal or intracerebroventricular administration (Csikós et al., 2023, Riquier and Sollars, 2019). Hence, microglia depletion creates a transient, receptive niche that permits donor cell engraftment (Shibuya et al., 2022, Xu et al., 2020, Xu et al., 2021). Although these depletion strategies may carry some toxicity risks such as mild liver enzyme abnormalities and hepatotoxicity, efforts are underway to optimize safety and improve clinical applicability (Tap et al., 2019, Capotondo et al., 2012).

In addition to pharmacological and chemotherapy-/irradiation-based approaches, genetic ablation offers precise temporal and cell-type-specific tools for studying microglial depletion and replacement approaches, while not directly translatable to clinical settings. For example, in CX3CR1^CreER^ × Rosa26^DTR^ mice, tamoxifen-induced DTR expression followed by diphtheria toxin injection selectively ablates microglia without affecting peripheral macrophage (Bennett et al., 2018; Lund et al., 2018). Another approach involves deletion of the *Csf1r* fms-intronic regulatory element (ΔFIRE), which generates viable microglia-deficient mice (Chadarevian et al., 2024). These efforts support the view that microglia play protective roles and offer mechanistic insights into replacement strategies.

### Circulation-derived Myeloid Cells Resemble Brain-resident Microglia

CDMCs have emerged as promising candidates for microglial replacement in AD. CDMCs are capable of engrafting into the CNS following host microglial depletion via CSF1R inhibition, or genetic ablation (Bennett and Bennett, 2020; Cronk et al., 2018; Shemer et al., 2018). Upon CNS entry, these cells adopt microglia-like morphologies and express core markers, yet they retain peripheral transcriptional and epigenetic signatures—including elevated expression of *Apoe, Ms4a7, Clec12a*, and *Mrc1*, and show altered responses to inflammatory stimuli (Bennett et al., 2018, Lund et al., 2018).

Pivotal studies have revealed that while other embryonically derived tissue macrophages, such as Kupffer cells, are faithfully replaced by HSCs-derived progenitors, bone marrow–derived microglia-like cells do not fully attain the YS-microglial identity. This deficiency is characterized by the absence of SALL1, a key regulator essential for microglial gene expression. However, recent studies, including our own, suggest that prolonged tissue residency of CDMCs may eventually reprogram brain macrophages to adopt embryonic-like states (Shibuya et al., 2022; Kim et al., 2025; Bastos et al., 2025). In line with this, another recent study reported that mouse fetal liver monocytes could develop into “*bona fide”* SALL1-positive microglia that were transcriptionally equivalent to their YS-derived counterparts (Bastos et al., 2025).

In the context of AD, CDMCs exhibit variable functional integration. Although they may exhibit limited plaque association and reduced expression of homeostatic genes, they can compensate for disease-relevant deficits under specific conditions. Specifically, Yoo et al. (2023) demonstrated that CDMCs expressing wild-type TREM2 effectively restored microglial function and alleviated multiple pathological features caused by TREM2 deficiency, including reduced Lamp1+ dystrophic neurites. These findings indicate that despite retaining transcriptional and epigenetic differences from resident microglia, CDMCs can fulfill key functional roles, highlighting their therapeutic potential for microglial replacement in AD.

Moreover, CDMCs are amenable to ex vivo gene editing and autologous transplantation, making them attractive for translational applications. However, because they originate from a YS-independent lineage, they may have limited capacity to fully recapitulate the homeostatic surveillance and developmental functions of resident microglia (Cronk et al., 2018, Shemer et al., 2018). Nevertheless, recent studies demonstrating that fetal monocytes can give rise to transcriptionally faithful microglia or “*bona fide* microglia” when exposed to the appropriate CNS environment, suggesting that this constraint can be mitigated (Bastos et al., 2025; Kim et al., 2025). Longitudinal, multiomic, and functional studies will be required to determine the durability and completeness of this transcriptional convergence, and to define the environmental and genetic factors that promote their integration and phenotypic fidelity.

### Human Pluripotent Stem Cell–derived Microglia-like Cells

Leveraging their human relevance, induced pluripotent stem cell (iPSC)- or human pluripotent stem cell–derived microglia-like cells are invaluable for modeling patient-specific genetic variants, offering a unique platform to unravel disease mechanisms and explore targeted therapeutic interventions for AD. iPSC-derived microglia (iMGs) have been functionally validated both in in vitro and in vivo using chimeric brain models and recapitulate key microglial features—migration, cytokine response, phagocytosis, and, upon Aβ exposure, upregulate clearance genes, such as *ABCA7*, *TREM2*, *CD33,* and *APOE* (Hasselmann et al., 2019, Mancuso et al., 2019). Notably, iMGs adopt plaque-associated morphologies and reduce amyloid pathology in AD models. For example, a recent study by Chadarevian et al. (2025) elegantly demonstrated that engineered iMGs expressing neprilysin under the control of a CD9 promoter not only exhibited improved brain engraftment but also significantly mitigated plaque burden and neuronal loss, suggesting that iMGs could also be a promising source for microglia-based cell therapy.

## DISCUSSION

Microglial replacement strategies employing microglia-like cells are emerging as a promising clinical approach for the treatment of AD and potentially other neurodegenerative conditions. While donor-derived cells can engraft into the brain and reduce amyloid pathology in AD mouse models, their ability to replicate the full spectrum of functions performed by YS-derived microglia, especially in aged or diseased settings, remains unresolved and warrants further investigation.

Preclinical studies have shown that engrafted cells can restore microglial function and alleviate disease pathology. In TREM2-deficient 5xFAD mice, wild-type CDMCs restored plaque compaction, reduced neuritic dystrophy, and restored TREM2-SYK signaling (Yoo et al., 2023), while HSCs-derived progenitors transplantation reduced Aβ burden and improved memory (Mishra et al., 2023). Similar benefits have been observed using Aβ-targeting chimeric–antigen receptor macrophages (Kim et al., 2024), and even in other neurodegeneration models such as frontotemporal dementia, adult-onset leukoencephalopathy with axonal spheroids and pigmented glia (ALSP), and Sandhoff disease, where replacement reversed lysosomal storage, restored lipid metabolism, and improved motor function (Colella et al., 2024, Wu et al., 2025, Tsourmas et al., 2024). However, the persistence and autonomy of endogenous microglia, as demonstrated by Füger et al. (2017), may pose a barrier to replacement. In this study, long-term in vivo imaging of individually labeled microglial cells revealed that brain-resident microglia are remarkably stable, exhibiting self-renewal with minimal replacement and estimated lifespans exceeding 22 to 29 months under homeostatic conditions in mice. Consistently, bone marrow transplantation alone results in limited replacement of resident microglia, reflecting their robust self-renewal and the stability of the CNS niche. This underscores the need for niche clearance strategies, such as CSF1R inhibition, to facilitate effective engraftment and integration of donor-derived cells (Ajami et al., 2007; Hashimoto et al., 2013). Additionally, aging alone can impair microglial phagocytosis and gene expression, indicating that the host environment critically shapes therapeutic outcomes (Silvin et al., 2022, Thomas et al., 2022).

Translation to human AD remains complex. Microglial gene signatures identified in AD brains (Friedman et al., 2018, Prater et al., 2023) show notable divergence from those in mouse models, including the expression of *CXCL14* and *CHI3L1*, and a range of partially homeostatic states that are not easily classified into canonical DAM categories. Whether reparative populations, such as those described by Tay et al. (2018) in mice, exist in human AD remains unknown. Moreover, determining whether microglial dysfunction is a primary driver of AD pathology, and identifying which patient populations would most benefit from microglial replacement therapy, remains an open challenge. While animal and postmortem studies support a central role for dysfunctional microglia in AD, definitive biomarkers of human microglial impairment are currently lacking.

Nonetheless, the finding that microglial dysfunction alone can drive neurodegeneration independently of amyloid pathology strengthens the rationale for cell-based therapeutic strategies. Socodato et al. (2020) demonstrated that deletion of RhoA, a small GTPase that regulates cytoskeletal dynamics critical for microglial morphology, process motility, and phagocytosis, led to widespread neural dysfunction. Loss of RhoA in adult microglia resulted in aberrant neural activation, excessive glutamate release, and TNF-α-mediated neurotoxicity, ultimately causing synaptic loss and memory impairment in the absence of Aβ deposition. Additional studies have similarly shown that selective impairments in microglial function can trigger neurodegeneration. For example, loss of TREM2, a receptor essential for lipid sensing and microglial activation, results in defective plaque compaction, synaptic degeneration, and cognitive decline (Wang et al., 2015, Ulland et al., 2017). Likewise, chronic activation of interferon signaling in aging or tauopathy models reprograms microglia toward a neurotoxic phenotype that accelerates synapse loss and neuronal death (Roy et al., 2020). These findings emphasize the essential role of microglial homeostasis in preserving neural circuit integrity and further support the therapeutic potential for microglia-targeted interventions, particularly cell replacement approaches.

In the absence of definitive biomarkers, microglial replacement strategies may be most appropriate for genetically defined AD subtypes with mutations in microglia-specific genes (eg, *TREM2*, *SORL1*, and *HAVCR2*), as illustrated in Figure 1. These approaches may also benefit patients with high neuroinflammatory signatures, where microglial dysfunction is likely to contribute to disease progression. Combining inflammatory markers in blood or cerebrospinal fluid with functional imaging and genetic screening could ultimately support more personalized microglia replacement therapies.

To date, no clinical trials have tested direct microglia replacement strategies using CSF1R inhibition and HSCT in AD patients. This reflects both technical barriers and a relatively recent shift toward viewing microglia as viable therapeutic targets. However, a recent Phase 2a trial of bone marrow–derived mesenchymal stem cell therapy (Laromestrocel; ClinicalTrials.gov: NCT05233774) showed potential to reduce brain atrophy and cognitive decline in AD patients (Rash et al., 2025), suggesting that cell-based therapies hold great promises. Notably, Wu et al. (2025) demonstrated that bone marrow transplantation could achieve near-complete microglia replacement and halt disease progression in ALSP mouse models and patients with CSF1R mutations. Although CSF1R inhibitors were not used, the underlying genetic deficiency is thought to have created a microglia-deficient niche that allowed efficient donor engraftment. While the precise mechanism remains unclear, the study provides strong evidence that microglial turnover and repopulation can be achieved in humans under certain pathological conditions. During a 24-month clinical follow-up, treated patients showed stable donor cell chimerism, improved neurological function, and no further radiographic progression. Additionally, preclinical studies in mouse models of Rett syndrome and Leigh syndrome have shown that HSCT can modulate disease outcomes (Derecki et al., 2012; Cronk et al., 2015; Nakai et al., 2024), raising the possibility that microglia replacement or reprogramming strategies may be applicable across a broader range of CNS disorders.

Despite encouraging progress, translating microglia replacement into clinical practice faces considerable challenges. The current reliance on preconditioning methods, such as chemotherapy or irradiation, may limit its applicability to a subset of neurological patients, underscoring the need for less invasive, transplantation-free approaches such as iMGs-like cells. Therefore, future efforts must prioritize minimizing toxicity and promoting functional integration of donor cells (Fig. 2C) to advance the field, especially considering the need for long-term evaluations to ascertain the functional resemblance of replaced cells to brain-resident microglia and to assess potential long-term side effects. In addition, gene-editing approaches hold promise for tailoring donor cells to resist inflammatory dysregulation or enhance tissue compatibility. Ultimately, therapeutic success will depend on targeting the right patient populations at the right timing (disease stage) to achieve safe and personalized treatment.

## Funding and Support

This work was supported and funded by 10.13039/100016275SNUH Lee Kun-hee Child Cancer & Rare Disease Project, Republic of Korea (grant number: 25B-001-0700).

## Author Contributions

**Yongjin Yoo:** Writing – review & editing, Supervision, Funding Acquisition, Conceptualization. **Jee Yoon Bang:** Writing – review & editing, Writing – original draft, Conceptualization.

## Declaration of Generative AI and AI-Assisted Technologies in the Writing Process

During the preparation of this work, OpenAI’s ChatGPT was used to assist with grammar and punctuation correction. The authors reviewed and edited the content as necessary and take full responsibility for the final version of the paper.

## Declaration of Competing Interests

The authors declare that they have no known competing financial interests or personal relationships that could have appeared to influence the work reported in this paper.

## References

[bib1] Abellanas M.A., Purnapatre M., Burgaletto C., Schwartz M. (2025). Monocyte-derived macrophages act as reinforcements when microglia fall short in Alzheimer’s disease. Nat Neurosci.

[bib2] Ajami B., Bennett J.L., Krieger C., Tetzlaff W., Rossi F.M. (2007). Local self-renewal can sustain CNS microglia maintenance and function throughout adult life. Nature neuroscience.

[bib3] Andrews S.J., Renton A.E., Fulton-Howard B., Podlesny-Drabiniok A., Marcora E., Goate A.M. (2023). The complex genetic architecture of Alzheimer’s disease: novel insights and future directions. EBioMedicine.

[bib4] Bastos J., O’Brien C., Vara-Pérez M., Mampay M., van Olst L., Barry-Carroll L., Kancheva D., Leduc S., Lievens A.L., Ali L. (2025). Monocytes can efficiently replace all brain macrophages and fetal liver monocytes can generate bona fide SALL1+ microglia. Immunity.

[bib5] Bellenguez C., Küçükali F., Jansen I.E., Kleineidam L., Moreno-Grau S., Amin N., Naj A.C., Campos-Martin R., Grenier-Boley B., Andrade V., Holmans P.A., Boland A., Damotte V., van der Lee, Costa M.R., Kuulasmaa T., Yang Q., de Rojas I., Bis J.C., Yaqub A., Lambert J.C. (2022). New insights into the genetic etiology of Alzheimer’s disease and related dementias. Nature genetics.

[bib6] Bennett F.C., Bennett M.L., Yaqoob F., Mulinyawe S.B., Grant G.A., Hayden Gephart M., Plowey E.D., Barres B.A. (2018). A Combination of Ontogeny and CNS Environment Establishes Microglial Identity. Neuron.

[bib7] Bennett M.L., Bennett F.C. (2020). The influence of environment and origin on brain resident macrophages and implications for therapy. Nat. Neurosci..

[bib8] Biber K., Neumann H., Inoue K., Boddeke H.W. (2007). Neuronal ‘On’ and ‘Off’ signals control microglia. Trends in neurosciences.

[bib9] Biffi A., Montini E., Lorioli L., Cesani M., Fumagalli F., Plati T., Baldoli C., Martino S., Calabria A., Canale S., Benedicenti F., Vallanti G., Biasco L., Leo S., Kabbara N., Zanetti G., Rizzo W.B., Mehta N.A., Cicalese M.P., Casiraghi M., Naldini L. (2013). Lentiviral hematopoietic stem cell gene therapy benefits metachromatic leukodystrophy. Science (New York, N.Y.).

[bib10] Butovsky O., Jedrychowski M.P., Moore C.S., Cialic R., Lanser A.J., Gabriely G., Koeglsperger T., Dake B., Wu P.M., Doykan C.E., Fanek Z., Liu L., Chen Z., Rothstein J.D., Ransohoff R.M., Gygi S.P., Antel J.P., Weiner H.L. (2014). Identification of a unique TGF-β-dependent molecular and functional signature in microglia. Nature neuroscience.

[bib11] Capotondo A., Milazzo R., Politi L.S., Quattrini A., Palini A., Plati T., Merella S., Nonis A., di Serio C., Montini E., Naldini L., Biffi A. Brain conditioning is instrumental for successful microglia reconstitution following hematopoietic stem cell transplantation Proceedings of the National Academy of Sciences of the United States of America 109 37 2012 15018 15023. https://doi.org/10.1073/pnas.1205858109. 10.1073/pnas.1205858109 PMC3443128 22923692

[bib12] Cartier N., Hacein-Bey-Abina S., Bartholomae C.C., Veres G., Schmidt M., Kutschera I., Vidaud M., Abel U., Dal-Cortivo L., Caccavelli L., Mahlaoui N., Kiermer V., Mittelstaedt D., Bellesme C., Lahlou N., Lefrère F., Blanche S., Audit M., Payen E., Leboulch P., Aubourg P. (2009). Hematopoietic stem cell gene therapy with a lentiviral vector in X-linked adrenoleukodystrophy. Science (New York, N.Y.).

[bib13] Chadarevian J.P., Davtyan H., Chadarevian A.L., Nguyen J., Capocchi J.K., Le L., Escobar A., Chadarevian T., Mansour K., Deynega E., Mgerian M., Tu C., Kiani Shabestari S., Carlen-Jones W., Eskandari-Sedighi G., Hasselmann J., Spitale R.C., Blurton-Jones M. (2025). Harnessing human iPSC-microglia for CNS-wide delivery of disease-modifying proteins. Cell stem cell.

[bib14] Chadarevian J.P., Hasselmann J., Lahian A., Capocchi J.K., Escobar A., Lim T.E., Le L., Tu C., Nguyen J., Kiani Shabestari S. (2024). Therapeutic potential of human microglia transplantation in a chimeric model of CSF1R-related leukoencephalopathy. Neuron.

[bib15] Clarke J.T., West M.L., Bultas J., Schiffmann R. (2007). The pharmacology of multiple regimens of agalsidase alfa enzyme replacement therapy for Fabry disease. Genetics in medicine : official journal of the American College of Medical Genetics.

[bib16] Colella P., Sayana R., Suarez-Nieto M.V., Sarno J., Nyame K., Xiong J., Pimentel Vera, Arozqueta Basurto J., Corbo M., Limaye A., Davis K.L., Abu-Remaileh M., Gomez-Ospina N. (2024). CNS-wide repopulation by hematopoietic-derived microglia-like cells corrects progranulin deficiency in mice. Nature communications.

[bib17] Corces, M.R., Shcherbina, A., Kundu, S., Gloudemans, M.J., Frésard, L., Granja, J.M., Louie, B.H., Eulalio, T., Shams, S., Bagdatli, S.T., Mumbach, M.R., Liu, B., Montine, K.S., Greenleaf, W.J., Kundaje, A., Montgomery, S.B., Chang, H.Y., & Montine, T.J. (2020). Single-cell epigenomic analyses implicate candidate causal variants at inherited risk loci for Alzheimer's and Parkinson's diseases. *Nature genetics*, *52*(11), 1158–1168. 10.1038/s41588-020-00721-x. PMC7606627 33106633

[bib18] Cronk J.C., Derecki N.C., Ji E., Xu Y., Lampano A.E., Smirnov I., Baker W., Norris G.T., Marin I., Coddington N., Wolf Y., Turner S.D., Aderem A., Klibanov A.L., Harris T.H., Jung S., Litvak V., Kipnis J. (2015). Methyl-CpG Binding Protein 2 Regulates Microglia and Macrophage Gene Expression in Response to Inflammatory Stimuli. Immunity.

[bib19] Cronk J.C., Filiano A.J., Louveau A., Marin I., Marsh R., Ji E., Goldman D.H., Smirnov I., Geraci N., Acton S., Overall C.C., Kipnis J. (2018). Peripherally derived macrophages can engraft the brain independent of irradiation and maintain an identity distinct from microglia. The Journal of experimental medicine.

[bib20] Csikós V., Oláh S., Dóra F., Arrasz N., Cservenák M., Dobolyi A. (2023). Microglia depletion prevents lactation by inhibition of prolactin secretion. iScience.

[bib21] Cugurra A., Mamuladze T., Rustenhoven J., Dykstra T., Beroshvili G., Greenberg Z.J., Baker W., Papadopoulos Z., Drieu A., Blackburn S., Kanamori M., Brioschi S., Herz J., Schuettpelz L.G., Colonna M., Smirnov I., Kipnis J. (2021). Skull and vertebral bone marrow are myeloid cell reservoirs for the meninges and CNS parenchyma. Science (New York, N.Y.).

[bib22] Dagher N.N., Najafi A.R., Kayala K.M., Elmore M.R., White T.E., Medeiros R., West B.L., Green K.N. (2015). Colony-stimulating factor 1 receptor inhibition prevents microglial plaque association and improves cognition in 3xTg-AD mice. Journal of neuroinflammation.

[bib23] Davalos D., Grutzendler J., Yang G. (2005). ATP mediates rapid microglial response to local brain injury *in vivo*. Nat Neurosci.

[bib24] Deczkowska A., Keren-Shaul H., Weiner A., Colonna M., Schwartz M., Amit I. (2018). Disease-Associated Microglia: A Universal Immune Sensor of Neurodegeneration. Cell.

[bib25] Derecki N.C., Cronk J.C., Lu Z., Xu E., Abbott S.B.G., Guyenet P.G., Kipnis J. (2012). Wild-type microglia arrest pathology in a mouse model of Rett syndrome. Nature.

[bib26] Du S., Drieu A., Cheng Y., Storck S.E., Rustenhoven J., Mamuladze T., Bhattarai B., Brioschi S., Nguyen K., Ou F., Cao J., Rodrigues P.F., Smirnov I., DeNardo D., Ginhoux F., Cella M., Colonna M., Kipnis (2024). Brain-engrafted monocyte-derived macrophages from blood and skull-bone marrow exhibit distinct identities from microglia. bioRxiv: the preprint server for biology.

[bib27] Dvir-Szternfeld R., Castellani G., Arad M., Cahalon L., Colaiuta S.P., Keren-Shaul H., Croese T., Burgaletto C., Baruch K., Ulland T., Colonna M., Weiner A., Amit I., Schwartz M. (2022). Alzheimer’s disease modification mediated by bone marrow-derived macrophages via a TREM2-independent pathway in mouse model of amyloidosis. Nature aging.

[bib28] Elmore M.R.P., Najafi A.R., Koike M.A., Dagher N.N., Spangenberg E.E., Rice R.A., Kitazawa M., Matusow B., Nguyen H., West B.L. (2014). Colony-stimulating factor 1 receptor signaling is necessary for microglia viability, unmasking a microglia progenitor cell in the adult brain. Neuron.

[bib29] Friedman B.A., Srinivasan K., Ayalon G., Meilandt W.J., Lin H., Huntley M.A., Cao Y., Lee S.H., Haddick P.C.G., Ngu H. (2018). Diverse brain myeloid expression profiles reveal distinct microglial activation states and aspects of Alzheimer’s disease not evident in mouse models. Cell Rep..

[bib30] Frisoni G.B., Altomare D., Thal D.R., Ribaldi F., van der Kant R., Ossenkoppele R., Blennow K., Cummings J., van Duijn C., Nilsson P.M. (2022). The probabilistic model of Alzheimer disease: the amyloid hypothesis revised. Nat. Rev. Neurosci..

[bib31] Füger P., Hefendehl J.K., Veeraraghavalu K., Wendeln A.C., Schlosser C., Obermüller U., Wegenast-Braun B.M., Neher J.J., Martus P., Kohsaka S. (2017). Microglia turnover with aging and in an Alzheimer’s model via long-term in vivo single-cell imaging. Nat. Neurosci..

[bib32] Fumagalli L., Nazlie Mohebiany A., Premereur J., Polanco Miquel P., Bijnens B., Van de Walle P., Fattorelli N., Mancuso R. (2025). Microglia heterogeneity, modeling and cell-state annotation in development and neurodegeneration. Nature neuroscience.

[bib33] Gao C., Jiang J., Tan Y. (2023). Microglia in neurodegenerative diseases: mechanism and potential therapeutic targets. *Sig Transduct Target*. Ther.

[bib34] Ginhoux F., Greter M., Leboeuf M., Nandi S., See P., Gokhan S., Mehler M.F., Conway S.J., Ng L.G., Stanley E.R., Samokhvalov I.M., Merad M. (2010). Fate mapping analysis reveals that adult microglia derive from primitive macrophages. Science (New York, N.Y.).

[bib35] Gjoneska E., Pfenning A.R., Mathys H., Quon G., Kundaje A., Tsai L.H., Kellis M. (2015). Conserved epigenomic signals in mice and humans reveal immune basis of Alzheimer’s disease. Nature.

[bib36] Goldmann T., Prinz M. (2013). Role of microglia in CNS autoimmunity. Clin Dev Immunol.

[bib37] Goldmann T, Wieghofer P, Jordão MJ, Prutek F, Hagemeyer N, Frenzel K, Amann L, Staszewski O, Kierdorf K, Krueger M, Locatelli G, Hochgerner H, Zeiser R, Epelman S, Geissmann F, Priller J, Rossi FM, Bechmann I, Kerschensteiner M, Linnarsson S, Jung S, Prinz M. Origin, fate and dynamics of macrophages at central nervous system interfaces. Nat Immunol. 2016 Jul;17(7):797-805. doi: 10.1038/ni.3423. 10.1038/ni.3423 PMC4968048 27135602

[bib38] Greenhalgh AD, Zarruk JG, Healy LM, Baskar Jesudasan SJ, Jhelum P, Salmon CK, Formanek A, Russo MV, Antel JP, McGavern DB, McColl BW, David S. Peripherally derived macrophages modulate microglial function to reduce inflammation after CNS injury. PLoS Biol. 2018 Oct 17;16(10):e2005264. doi: 10.1371/journal.pbio.2005264. PMID: 30332405; PMCID: PMC6205650. 10.1371/journal.pbio.2005264 PMC6205650 30332405

[bib39] Grubman A., Vandekolk T.H., Schröder J., Sun G., Hatwell-Humble J., Chan J., Oksanen M., Lehtonen S., Hunt C., Koistinaho J.E., Nilsson S.K., Haynes J.M., Pouton C.W., Schröder J.M. (2020). A CX3CR1 Reporter hESC Line Facilitates Integrative Analysis of In-Vitro-Derived Microglia and Improved Microglia Identity upon Neuron-Glia Co-culture. Stem Cell Reports.

[bib40] Guan F., Wang R., Yi Z., Luo P., Liu W., Xie Y., Liu Z., Xia Z., Zhang H., Cheng Q. (2025). Tissue macrophages: origin, heterogenity, biological functions, diseases and therapeutic targets. Signal Transduction and Targeted Therapy.

[bib41] Guerreiro R., Wojtas A., Bras J., Carrasquillo M., Rogaeva E., Majounie E., Cruchaga C., Sassi C., Kauwe J.S.K., Younkin S. (2013). TREM2 variants in Alzheimer’s disease. N. Engl. J. Med..

[bib42] Hashimoto D., Chow A., Noizat C., Teo P., Beasley M.B., Leboeuf M., Becker C.D., See P., Price J., Lucas D., Greter M., Mortha A., Boyer S.W., Forsberg E.C., Tanaka M., van Rooijen N., García-Sastre A., Stanley E.R., Ginhoux F., Frenette P.S., Merad M. (2013). Tissue-resident macrophages self-maintain locally throughout adult life with minimal contribution from circulating monocytes. Immunity.

[bib43] Hasselmann J., Coburn M.A., England W., Velez D.X.F., Shabestari S.K., Tu C.H., McQuade A., Kolahdouzan M., Echeverria K., Claes C., Nakayama T., Azevedo R., Coufal N.G., Han C.Z., Cummings B.J., Davtyan H., Glass C.K., Healy L.M., Gandhi S.P., Blurton-Jones M. (2019). Development of a chimeric model to study and manipulate human microglia in vivo. Neuron.

[bib44] Haynes S.E., Hollopeter G., Yang G., Kurpius D., Dailey M.E., Gan W.B., Julius D. (2006). The P2Y12 receptor regulates microglial activation by extracellular nucleotides. Nat. Neurosci..

[bib45] Herisson F., Frodermann V., Courties G., Rohde D., Sun Y., Vandoorne K., Wojtkiewicz G.R., Masson G.S., Vinegoni C., Kim J., Kim D., Weissleder R., Swirski F.K., Moskowitz M.A., Nahrendorf M. (2018). Direct vascular channels connect skull bone marrow and the brain surface enabling myeloid cell migration. Nature Neuroscience.

[bib46] Hong S., Beja-Glasser V.F., Nfonoyim B.M., Frouin A., Li S., Ramakrishnan S., Merry K.M., Shi Q., Rosenthal A., Barres B.A., Lemere C.A., Selkoe D.J., Stevens B. (2016). Complement and microglia mediate early synapse loss in Alzheimer mouse models. Science.

[bib47] Jansen I.E., Savage J.E., Watanabe K., Bryois J., Williams D.M., Steinberg S., Sealock J., Karlsson I.K., Hägg S., Athanasiu L., Voyle N., Proitsi P., Witoelar A., Stringer S., Aarsland D., Almdahl I.S., Andersen F., Bergh S., Bettella F., Posthuma D. (2019). Genome-wide meta-analysis identifies new loci and functional pathways influencing Alzheimer’s disease risk. Nature Genetics.

[bib48] Keren-Shaul H., Spinrad A., Weiner A., Matcovitch-Natan O., Dvir-Szternfeld R., Ulland T.K., David E., Baruch K., Lara-Astaiso D., Toth B., Itzkovitz S., Colonna M., Schwartz M., Amit I. (2017). A Unique Microglia Type Associated with Restricting Development of Alzheimer’s Disease. Cell.

[bib49] Kierdorf K., Erny D., Goldmann T. (2013). Microglia emerge from erythromyeloid precursors via Pu.1- and Irf8-dependent pathways. Nat Neurosci.

[bib50] Kierdorf K., Prinz M. (2013). Factors regulating microglia activation. Frontiers in cellular neuroscience.

[bib51] Kim J.S., Trzebanski S., Shin S.H., Schori L., Frumer Friedman, Ilani N.C., Kadam A., Vicario R., Aust O., Bugaeva P., Piatek S., Ismajli L.K., Hoffmann C.J., Scheller M., Boura-Halfon S., Kaushansky N., Golani O., Solomon A., Liu Z., Amann L., Jung S. (2025). Clonal hematopoiesis-associated motoric deficits caused by monocyte-derived microglia accumulating in aging mice. Cell reports.

[bib52] Kim A.B., Xiao Q., Yan P., Pan Q., Pandey G., Grathwohl S., Gonzales E., Xu I., Cho Y., Haecker H., Epelman S., Diwan A., Lee J., DeSelm C.J. (2024). Chimeric antigen receptor macrophages target and resorb amyloid plaques. JCI Insight.

[bib53] Korczyn A.D., Grinberg L.T. (2024). Is Alzheimer disease a disease?. Nat. Rev. Neurol..

[bib54] Kosoy R., Fullard J.F., Zeng B., Bendl J., Dong P., Rahman S., Kleopoulos S.P., Shao Z., Girdhar K., Humphrey J. (2022). Genetics of the human microglia regulome refines Alzheimer’s disease risk loci. Nat. Genet..

[bib55] Krasemann S., Madore C., Cialic R., Baufeld C., Calcagno N., Fatimy R.E., Beckers L., O’Loughlin E., Xu Y., Fanek Z., Greco D.J., Smith S.T., Tweet G., Humulock Z., Zrzavy T., Conde-Sanroman P., Gacias M., Weng Z., Chen H., Butovsky O. (2017). The TREM2-APOE pathway drives the transcriptional phenotype of dysfunctional microglia in neurodegenerative diseases. Immunity.

[bib56] Kunkle B.W., Grenier-Boley B., Sims R., Bis J.C., Damotte V., Naj A.C., Boland A., Vronskaya M., Van Der Lee, Amlie-Wolf A., Bellenguez C., Frizatti A., Chouraki V., Martin E.R., Sleegers K., Badarinarayan N., Jakobsdottir J., Hamilton-Nelson K.L., Moreno-Grau S., Pericak-Vance M.A. (2019). Genetic meta-analysis of diagnosed Alzheimer’s disease identifies new risk loci and implicates Aβ, tau, immunity and lipid processing. Nature Genetics.

[bib57] Kunkle B.W., Schmidt M., Klein H., Naj A.C., Hamilton-Nelson K.L., Larson E.B., Evans D.A., De Jager, Crane P.K., Buxbaum J.D., Ertekin-Taner N., Barnes L.L., Fallin M.D., Manly J.J., Go R.C.P., Obisesan T.O., Kamboh M.I., Bennett D.A., Hall K.S., Kukull W.A. (2020). Novel Alzheimer Disease Risk LOCI and pathways in African American individuals using the African Genome Resources Panel. JAMA Neurology.

[bib58] Lambert J., Ibrahim-Verbaas C.A., Harold D., Naj A.C., Sims R., Bellenguez C., Jun G., DeStefano A.L., Bis J.C., Beecham G.W., Grenier-Boley B., Russo G., Thornton-Wells T.A., Jones N., Smith A.V., Chouraki V., Thomas C., Ikram M.A., Zelenika D., Amouyel P. (2013). Meta-analysis of 74,046 individuals identifies 11 new susceptibility loci for Alzheimer’s disease. Nature Genetics.

[bib59] Li Q., Barres B.A. (2018). Microglia and macrophages in brain homeostasis and disease. Nat. Rev. Immunol..

[bib60] Liberzon A., Birger C., Thorvaldsdóttir H., Ghandi M., Mesirov J.P., Tamayo P. (2015). The Molecular Signatures Database Hallmark Gene Set Collection. Cell Systems.

[bib61] Liu Y., Zhou L., Wang J., Li D., Ren W., Peng J., Wei X., Xu T., Xin W., Pang R., Li Y., Qin Z., Murugan M., Mattson M.P., Wu L., Liu X. (2016). TNF-α Differentially Regulates Synaptic Plasticity in the Hippocampus and Spinal Cord by Microglia-Dependent Mechanisms after Peripheral Nerve Injury. Journal of Neuroscience.

[bib62] Long J.M., Holtzman D.M. (2019). Alzheimer disease: an update on pathobiology and treatment strategies. Cell.

[bib63] Lund H., Pieber M., Parsa R., Han J., Grommisch D., Ewing E., Kular L., Needhamsen M., Espinosa A., Nilsson E., Överby A.K., Butovsky O., Jagodic M., Zhang X., Harris R.A. (2018). Competitive repopulation of an empty microglial niche yields functionally distinct subsets of microglia-like cells. Nature Communications.

[bib64] Mancuso R., Van Den Daele J., Fattorelli N., Wolfs L., Balusu S., Burton O., Liston A., Sierksma A., Fourne Y., Poovathingal S. (2019). Stem-cell-derived human microglia transplanted in mouse brain to study human disease. Nat. Neurosci..

[bib65] Marioni R.E., Harris S.E., Zhang Q., McRae A.F., Hagenaars S.P., Hill W.D., Davies G., Ritchie C.W., Gale C.R., Starr J.M., Goate A.M., Porteous D.J., Yang J., Evans K.L., Deary I.J., Wray N.R., Visscher P.M. (2018). GWAS on family history of Alzheimer’s disease. Translational Psychiatry.

[bib66] Marschallinger J., Iram T., Zardeneta M., Lee S.E., Lehallier B., Haney M.S., Pluvinage J.V., Mathur V., Hahn O., Morgens D.W., Kim J., Tevini J., Felder T.K., Wolinski H., Bertozzi C.R., Bassik M.C., Aigner L., Wyss-Coray T. (2020). Lipid-droplet-accumulating microglia represent a dysfunctional and proinflammatory state in the aging brain. Nature Neuroscience.

[bib67] Mass E., Nimmerjahn F., Kierdorf K., Schlitzer A. (2023). Tissue-specific macrophages: how they develop and choreograph tissue biology. Nat. Rev. Immunol..

[bib68] Masuda T., Sankowski R., Staszewski O., Prinz M. (2020). Microglia heterogeneity in the Single-Cell era. Cell Reports.

[bib69] Masuda T., Sankowski R., Staszewski O., Prinz M. (2020). Microglia heterogeneity in the Single-Cell era. Cell Reports.

[bib70] Mathys H., Adaikkan C., Gao F., Young J.Z., Manet E., Hemberg M., De Jager P.L., Ransohoff R.M., Regev A., Tsai L.H. (2017). Temporal tracking of microglia activation in neurodegeneration at single-cell resolution. Cell Rep..

[bib71] Mathys H., Davila-Velderrain J., Peng Z., Gao F., Mohammadi S., Young J.Z., Menon M., He L., Abdurrob F., Jiang X., Martorell A.J., Ransohoff R.M., Hafler B.P., Bennett D.A., Kellis M., Tsai L. (2019). Single-cell transcriptomic analysis of Alzheimer’s disease. Nature.

[bib72] Mawuenyega K.G., Sigurdson W., Ovod V., Munsell L., Kasten T., Morris J.C., Yarasheski K.E., Bateman R.J. (2010). Decreased clearance of CNS Β-Amyloid in Alzheimer’s disease. Science.

[bib73] Mildner A., Huang H., Radke J., Stenzel W., Priller J. (2016). P2Y12receptor is expressed on human microglia under physiological conditions throughout development and is sensitive to neuroinflammatory diseases. Glia.

[bib74] Mishra P., Silva A., Sharma J., Nguyen J., Pizzo D.P., Hinz D., Sahoo D., Cherqui S. (2023). Rescue of Alzheimer’s disease phenotype in a mouse model by transplantation of wild-type hematopoietic stem and progenitor cells. Cell reports.

[bib75] Mootha V.K., Lindgren C.M., Eriksson K., Subramanian A., Sihag S., Lehar J., Puigserver P., Carlsson E., Ridderstråle M., Laurila E., Houstis N., Daly M.J., Patterson N., Mesirov J.P., Golub T.R., Tamayo P., Spiegelman B., Lander E.S., Hirschhorn J.N., Groop L.C. (2003). PGC-1α-responsive genes involved in oxidative phosphorylation are coordinately downregulated in human diabetes. Nature Genetics.

[bib76] Muñoz-Castro, C., Mejias-Ortega, M., Sanchez-Mejias, E., Navarro, V., Trujillo-Estrada, L., Jimenez, S., Garcia-Leon, J. A., Fernandez-Valenzuela, J. J., Sanchez-Mico, M. V., Romero-Molina, C., Moreno-Gonzalez, I., Baglietto-Vargas, D., Vizuete, M., Gutierrez, A., & Vitorica, J. (2023). Monocyte-derived cells invade brain parenchyma and amyloid plaques in human Alzheimer's disease hippocampus. *Acta neuropathologica communications*, *11*(1), 31. 10.1186/s40478-023-01530-z. PMC9976401 36855152

[bib77] Nakai R., Varnum S., Field R.L., Shi H., Giwa R., Jia W., Krysa S.J., Cohen E.F., Borcherding N., Saneto R.P. (2024). Mitochondria transfer-based therapies reduce the morbidity and mortality of Leigh syndrome. Nat. Metab..

[bib78] Nakanishi H., Ni J., Nonaka S., Hayashi Y. (2021). Microglial circadian clock regulation of microglial structural complexity, dendritic spine density and inflammatory response. Neurochemistry international.

[bib79] Nimmerjahn A., Kirchhoff F., Helmchen F. (2005). Resting microglial cells are highly dynamic surveillants of brain parenchyma in vivo. Science (New York, N.Y.).

[bib80] Nott A., Holtman I.R., Coufal N.G., Schlachetzki J.C.M., Yu M., Hu R., Han C.Z., Pena M., Xiao J., Wu Y., Keulen Z., Pasillas M.P., O’Connor C., Nickl C.K., Schafer S.T., Shen Z., Rissman R.A., Brewer J.B., Gosselin D., Glass C.K. (2019). Brain cell type–specific enhancer–promoter interactome maps and disease - risk association. Science.

[bib81] Novikova G., Kapoor M., Tcw J., Abud E.M., Efthymiou A.G., Chen S.X., Cheng H., Fullard J.F., Bendl J., Liu Y., Roussos P., Björkegren J.L., Liu Y., Poon W.W., Hao K., Marcora E., Goate A.M. (2021). Integration of Alzheimer’s disease genetics and myeloid genomics identifies disease risk regulatory elements and genes. Nature communications.

[bib82] Olah M., Menon V., Habib N., Taga M.F., Ma Y., Yung C.J., Cimpean M., Khairallah A., Coronas-Samano G., Sankowski R., Grün D., Kroshilina A.A., Dionne D., Sarkis R.A., Cosgrove G.R., Helgager J., Golden J.A., Pennell P.B., Prinz M., Vonsattel J.P.G., De Jager P.L. (2020). Single cell RNA sequencing of human microglia uncovers a subset associated with Alzheimer’s disease. Nature communications.

[bib83] Paolicelli R.C., Bolasco G., Pagani F., Maggi L., Scianni M., Panzanelli P., Giustetto M., Ferreira T.A., Guiducci E., Dumas L., Ragozzino D., Gross C.T. (2011). Synaptic pruning by microglia is necessary for normal brain development. Science (New York, N.Y.).

[bib84] Paolicelli R.C., Sierra A., Stevens B., Tremblay M.E., Aguzzi A., Ajami B., Amit I., Audinat E., Bechmann I., Bennett M., Bennett F., Bessis A., Biber K., Bilbo S., Blurton-Jones M., Boddeke E., Brites D., Brône B., Brown G.C., Butovsky O., Wyss-Coray T. (2022). Microglia states and nomenclature: A field at its crossroads. Neuron.

[bib85] Prater K.E., Green K.J., Mamde S., Sun W., Cochoit A., Smith C.L., Chiou K.L., Heath L., Rose S.E., Wiley J. (2023). Human microglia show unique transcriptional changes in Alzheimer’s disease. Nat. Aging.

[bib86] Prinz M., Jung S., Priller J. (2019). Microglia biology: one century of evolving concepts. Cell.

[bib87] Rachmian N., Medina S., Cherqui U., Akiva H., Deitch D., Edilbi D., Croese T., Salame T.M., Ramos J.M.P., Cahalon L. (2024). Identification of senescent, TREM2-expressing microglia in aging and Alzheimer’s disease model mouse brain. Nat. Neurosci..

[bib88] Rash B.G., Ramdas K.N., Agafonova N., Naioti E., McClain-Moss L., Zainul Z., Varnado B., Peterson K., Brown M., Leal T. (2025). Allogeneic mesenchymal stem cell therapy with laromestrocel in mild Alzheimer’s disease: a randomized controlled phase 2a trial. Nat. Med..

[bib89] Ransohoff R.M., El Khoury J. (2015). Microglia in Health and Disease. Cold Spring Harbor perspectives in biology.

[bib90] Rao Y., Peng B. (2024). Allogenic microglia replacement: a novel therapeutic strategy for neurological disorders. Fundam. Res..

[bib91] Riquier A.J., Sollars S.I. (2019). Astrocytic response to neural injury is larger during development than in adulthood and is not predicated upon the presence of microglia. Brain, behavior, & immunity - health.

[bib92] Roy E.R., Wang B., Wan Y.W., Chiu G., Cole A., Yin Z., Propson N.E., Xu Y., Jankowsky J.L., Liu Z., Lee V.M., Trojanowski J.Q., Ginsberg S.D., Butovsky O., Zheng H., Cao W. (2020). Type I interferon response drives neuroinflammation and synapse loss in Alzheimer disease. The Journal of clinical investigation.

[bib93] Salter M.W., Stevens B. (2017). Microglia emerge as central players in brain disease. Nat. Med..

[bib94] Schafer D.P., Lehrman E.K., Kautzman A.G., Koyama R., Mardinly A.R., Yamasaki R., Ransohoff R.M., Greenberg M.E., Barres B.A., Stevens B. (2012). Microglia sculpt postnatal neural circuits in an activity and complement-dependent manner. Neuron.

[bib95] Scheltens P., De Strooper B., Kivipelto M., Holstege H., Chételat G., Teunissen C.E., Cummings J., van der Flier W.M. (2021). Alzheimer’s disease. Lancet.

[bib96] Schiffmann R., Kopp J.B., Austin H.A., Sabnis S., Moore D.F., Weibel T., Balow J.E., Brady R.O. (2001). Enzyme replacement therapy in Fabry disease: a randomized controlled trial. JAMA.

[bib97] Selkoe D.J., Hardy J. (2016). The amyloid hypothesis of Alzheimer’s disease at 25 years. EMBO molecular medicine.

[bib98] Sellgren C.M., Gracias J., Watmuff B., Biag J.D., Thanos J.M., Whittredge P.B., Fu T., Worringer K., Brown H.E., Wang J. (2019). Increased synapse elimination by microglia in schizophrenia patient-derived models of synaptic pruning. Nat. Neurosci..

[bib99] Shemer A., Grozovski J., Tay T.L., Tao J., Volaski A., Süß P., Ardura-Fabregat A., Gross-Vered M., Kim J.S., David E., Chappell-Maor L., Thielecke L., Glass C.K., Cornils K., Prinz M., Jung S. (2018). Engrafted parenchymal brain macrophages differ from microglia in transcriptome, chromatin landscape and response to challenge. Nature communications.

[bib100] Shibuya Y., Kumar K.K., Mader M.M., Yoo Y., Ayala, Zhou M., Mohr M.A., Neumayer G., Kumar I., Yamamoto R., Marcoux P., Liou B., Bennett F.C., Nakauchi H., Sun Y., Chen X., Heppner F.L., Wyss-Coray T., Südhof T.C., Wernig M. (2022). Treatment of a genetic brain disease by CNS-wide microglia replacement. Science translational medicine.

[bib101] Sierra A., Encinas J.M., Deudero J.J.P., Chancey J.H., Enikolopov G., Overstreet-Wadiche L.S., Tsirka S.E., Maletic-Savatic M. (2010). Microglia shape adult hippocampal neurogenesis through apoptosis-coupled phagocytosis. Cell Stem Cell.

[bib102] Silvin A., Uderhardt S., Piot C., Da Mesquita S., Yang K., Geirsdottir L., Mulder K., Eyal D., Liu Z., Bridlance C. (2022). Dual ontogeny of disease-associated microglia and disease inflammatory macrophages in aging and neurodegeneration. Immunity.

[bib103] Socodato R., Portugal C.C., Canedo T., Rodrigues A., Almeida T.O., Henriques J.F., Vaz S.H., Magalhães J., Silva C.M., Baptista F.I., Alves R.L., Coelho-Santos V., Silva A.P., Paes-de-Carvalho R., Magalhães A., Brakebusch C., Sebastião A.M., Summavielle T., Ambrósio A.F., Relvas J.B. (2020). Microglia Dysfunction Caused by the Loss of Rhoa Disrupts Neuronal Physiology and Leads to Neurodegeneration. Cell reports.

[bib104] Sonoda H., Morimoto H., Yoden E., Koshimura Y., Kinoshita M., Golovina G., Takagi H., Yamamoto R., Minami K., Mizoguchi A., Tachibana K., Hirato T., Takahashi K. (2018). A Blood-Brain-Barrier-Penetrating Anti-human Transferrin Receptor Antibody Fusion Protein for Neuronopathic Mucopolysaccharidosis II. Molecular therapy : the journal of the American Society of Gene Therapy.

[bib105] Spangenberg E.E., Lee R.J., Najafi A.R., Rice R.A., Elmore M.R., Blurton-Jones M., West B.L., Green K.N. (2016). Eliminating microglia in Alzheimer’s mice prevents neuronal loss without modulating amyloid-β pathology. Brain : a journal of neurology.

[bib106] Stevens B., Allen N.J., Vazquez L.E., Howell G.R., Christopherson K.S., Nouri N., Micheva K.D., Mehalow A.K., Huberman A.D., Stafford B., Sher A., Litke A.M., Lambris J.D., Smith S.J., John S.W., Barres B.A. (2007). The classical complement cascade mediates CNS synapse elimination. Cell.

[bib107] Subramanian A., Tamayo P., Mootha V.K., Mukherjee S., Ebert B.L., Gillette M.A., Paulovich A., Pomeroy S.L., Golub T.R., Lander E.S., Mesirov J.P. (2005). Gene set enrichment analysis: a knowledge-based approach for interpreting genome-wide expression profiles. Proceedings of the National Academy of Sciences of the United States of America.

[bib108] Sun N., Victor M.B., Park Y.P., Xiong X., Scannail A.N., Leary N., Prosper S., Viswanathan S., Luna X., Boix C.A. (2023). Human microglial state dynamics in Alzheimer’s disease progression. Cell.

[bib109] Tap W.D., Gelderblom H., Palmerini E., Desai J., Bauer S., Blay J.Y., Alcindor T., Ganjoo K., Martín-Broto J., Ryan C.W., Thomas D.M., Peterfy C., Healey J.H., van de Sande M., Gelhorn H.L., Shuster D.E., Wang Q., Yver A., Hsu H.H., Lin P.S., ENLIVEN (2019). Pexidartinib versus placebo for advanced tenosynovial giant cell tumour (ENLIVEN): a randomised phase 3 trial. Lancet (London, England).

[bib110] Tay, T. L., Sagar, Dautzenberg, J., Grün, D., & Prinz, M. (2018). Unique microglia recovery population revealed by single-cell RNAseq following neurodegeneration. *Acta neuropathologica communications*, *6*(1), 87. 10.1186/s40478-018-0584-3. PMC6123921 30185219

[bib111] Thomas A.L., Lehn M.A., Janssen E.M., Hildeman D.A., Chougnet C.A. (2022). Naturally-aged microglia exhibit phagocytic dysfunction accompanied by gene expression changes reflective of underlying neurologic disease. Scientific reports.

[bib112] Tsourmas, K. I., Butler, C. A., Kwang, N. E., Sloane, Z. R., Dykman, K. J. G., Maloof, G. O., Prekopa, C. A., Krattli, R. P., El-Khatib, S. M., Swarup, V., Acharya, M. M., Hohsfield, L. A., & Green, K. N. (2024). Myeloid-derived β-hexosaminidase is essential for neuronal health and lysosome function: implications for Sandhoff disease. *bioRxiv : the preprint server for biology*, 2024.10.21.619538. 10.1101/2024.10.21.619538.

[bib113] Ulland T.K., Song W.M., Huang S.C.C., Ulrich J.D., Sergushichev A., Beatty W.L., Loboda A.A., Zhou Y., Cairns N.J., Kambal A. (2017). TREM2 maintains microglial metabolic fitness in Alzheimer’s disease. Cell.

[bib114] Varvel N.H., Grathwohl S.A., Baumann F., Liebig C., Bosch A., Brawek B., Thal D.R., Charo I.F., Heppner F.L., Aguzzi A., Garaschuk O., Ransohoff R.M., Jucker M. (2012). Microglial repopulation model reveals a robust homeostatic process for replacing CNS myeloid cells. Proceedings of the National Academy of Sciences of the United States of America.

[bib115] Walsh A.E., Lukens J.R. (2025). Harnessing microglia-based cell therapies for the treatment of neurodegenerative diseases. Current opinion in immunology.

[bib116] Wang Y., Cella M., Mallinson K., Ulrich J.D., Young K.L., Robinette M.L., Gilfillan S., Krishnan G.M., Sudhakar S., Zinselmeyer B.H. (2015). TREM2 lipid sensing sustains the microglial response in an Alzheimer’s disease model. Cell.

[bib117] Wang C., Fan L., Khawaja R.R., Liu B., Zhan L., Kodama L., Chin M., Li Y., Le D., Zhou Y., Condello C., Grinberg L.T., Seeley W.W., Miller B.L., Mok S.A., Gestwicki J.E., Cuervo A.M., Luo W., Gan L. (2022). Microglial NF-κB drives tau spreading and toxicity in a mouse model of tauopathy. Nature communications.

[bib118] Wightman D.P., Jansen I.E., Savage J.E., Shadrin A.A., Bahrami S., Holland D., Rongve A., Børte S., Winsvold B.S., Drange O.K., Martinsen A.E., Skogholt A.H., Willer C., Bråthen G., Bosnes I., Nielsen J.B., Fritsche L.G., Thomas L.F., Pedersen L.M., Gabrielsen M.E., Posthuma D. (2022). Author Correction: A genome-wide association study with 1,126,563 individuals identifies new risk loci for Alzheimer’s disease. Nature genetics.

[bib119] Wightman D.P., Jansen I.E., Savage J.E., Shadrin A.A., Bahrami S., Holland D., Rongve A., Børte S., Winsvold B.S., Drange O.K., Martinsen A.E., Skogholt A.H., Willer C., Bråthen G., Bosnes I., Nielsen J.B., Fritsche L.G., Thomas L.F., Pedersen L.M., Gabrielsen M.E., Posthuma D. (2021). A genome-wide association study with 1,126,563 individuals identifies new risk loci for Alzheimer’s disease. Nature genetics.

[bib120] Wu J., Wang Y., Li X., Ouyang P., Cai Y., He Y., Zhang M., Luan X., Jin Y., Wang J., Xiao Y., Liang Y., Xie F., Shu Y., Hu J., Chang C., Jiang J., Wu D., Zhao Y., Liu T., Peng B. (2025). Microglia replacement halts the progression of microgliopathy in mice and humans. Science (New York, N.Y.).

[bib121] Yoo Y., Neumayer G., Shibuya Y., Marc-Daniel Mader M., Wernig M. (2023). A cell therapy approach to restore microglial Trem2 function in a mouse model of Alzheimer’s disease. Cell Stem Cell.

[bib122] Xu Z., Rao Y., Huang Y., Zhou T., Feng R., Xiong S., Yuan T.F., Qin S., Lu Y., Zhou X., Li X., Qin B., Mao Y., Peng B. (2020). Efficient Strategies for Microglia Replacement in the Central Nervous System. Cell reports.

[bib123] Xu Z., Zhou X., Peng B., Rao Y. (2021). Microglia replacement by bone marrow transplantation (Mr BMT) in the central nervous system of adult mice. STAR protocols.

[bib124] Zhan Y., Paolicelli R.C., Sforazzini F., Weinhard L., Bolasco G., Pagani F., Vyssotski A.L., Bifone A., Gozzi A., Ragozzino D., Gross C.T. (2014). Deficient neuron-microglia signaling results in impaired functional brain connectivity and social behavior. Nature neuroscience.

[bib125] Zhang Y., Sloan S.A., Clarke L.E., Caneda C., Plaza C.A., Blumenthal P.D., Vogel H., Steinberg G.K., Edwards M.S., Li G., Duncan J.A., Cheshier S.H., Shuer L.M., Chang E.F., Grant G.A., Gephart M.G., Barres B.A. (2016). Purification and Characterization of Progenitor and Mature Human Astrocytes Reveals Transcriptional and Functional Differences with Mouse. Neuron.

